# Characterizing the relationship between functional network dynamics and the body mass index

**DOI:** 10.3389/fnut.2026.1734850

**Published:** 2026-04-14

**Authors:** Xiaoyang Xin, Jing Wang, Binghong Chen, Ying Li, Yongquan Huo

**Affiliations:** 1 School of Psychology, Shaanxi Normal University, Xi’an, Shaanxi, China; 2 Preschool College, Luoyang Normal University, Luoyang, China

**Keywords:** body mass index, dynamic functional connectivity, graph theory, independent component analysis, resting-state fMRI

## Abstract

**Background:**

Obesity has increasingly been linked to alterations in brain functional network organization. While static functional connectivity studies have revealed important associations with body mass index (BMI), the role of dynamic functional network properties and their topological variability remains largely unexplored. This study aimed to investigate the relationship between BMI and the dynamics of brain functional network connectivity.

**Methods:**

Resting-state fMRI data from 776 healthy young adults were analyzed using independent component analysis to extract independent components (ICs) and a sliding-window approach to derive dynamic brain connectivity metrics. Four recurring whole-brain functional connectivity states were identified, and the temporal variability of topological properties was calculated. We examined the relationships between BMI and the temporal properties of FC states, and between BMI and the temporal variability of topological properties.

**Results:**

In the whole-brain level, BMI was positively correlated with the fractional window and mean dwell time of one FC state, which was characterized by strong intra-network and inter-network connectivity of the visual network (VN). In the regional level, significant negative correlations were found between BMI and the temporal variability of nodal efficiency and local efficiency in ICs belonging to the VN.

**Conclusion:**

Our findings suggest modest, statistically significant associations between BMI and whole-brain functional network dynamics, with VN-dominant connectivity patterns emerging as candidate correlates of higher BMI.

## 1 Introduction

Obesity, characterized by excessive adipose tissue accumulation and abnormal body weight due to an imbalance between energy intake and expenditure, has emerged as a critical global public health challenge. On 4 March 2025, the World Obesity Atlas 2025 released by the World Obesity Federation predicts that the number of adults living with obesity worldwide will grow from 524 million in 2010 to 1.13 billion by 2030—an increase of over 115%. Its prevalence has nearly tripled since 1975 and continues to rise worldwide (1). Body mass index (BMI)—calculated as weight in kilograms divided by height in meters squared (kg/m^2^)—remains the most widely used proxy for adiposity in both epidemiological and neuroimaging research. Although BMI does not distinguish between fat and lean mass, it provides a standardized, non-invasive, and internationally accepted index of obesity-related health risk (2, 3). Notably, BMI not only serves as a practical measure of obesity risk at the population level but also offers a critical entry point for neuroimaging studies aiming to uncover the neural mechanisms underlying obesity.

Resting-state fMRI (rs-fMRI) is a powerful tool for elucidating the neural mechanisms underlying obesity, providing network-level insights into how excess body weight may alter brain organization. Neuroimaging evidence has consistently shown associations between BMI and brain network organization. In particular, studies using static functional connectivity (sFC) have demonstrated aberrant interactions within and between the default mode network (DMN), salience network (SN), and executive control network (ECN) in relation to BMI (4, 5). These alterations—often characterized by reduced intra-network coherence and impaired inter-network integration—are thought to reflect weakened top-down control and heightened sensitivity to reward-related cues (5). Importantly, recent work has also highlighted abnormal connectivity patterns within visual regions, suggesting that sensory-perceptual systems may play a critical role in linking BMI with maladaptive cognitive and affective processing. However, majority of prior studies adopted conventional *static* connectivity measures that assume functional connectivity is constant during entire scan.

The resting brain is a highly dynamic system with non-stationary neural activity and rapidly changing neural interactions, which contains meaningful neural information that cannot be captured by the conventional ‘static’ brain connectivity analytic approach (6, 7). For example, measures of the temporal variability of resting-state dFC in certain brain regions have been linked to various clinical conditions (8–10), and a recent study reported that BMI was correlated with the dFC variability of regions in the limbic and visual networks (11).

Beyond examining variability in specific connections or regions, a series of dynamic brain studies have demonstrated that the time-varying features of whole-brain dFC can be described by a series of reoccurring whole-brain FC patterns, regarded as “FC states.” The brain spontaneously transitions among a set of discrete FC states, and particular states have been associated with specific cognitive or mental processes (12–14). Using such a state-based analysis, Tan et al. (15) found that BMI was positively correlated with the mean dwell time in one FC state characterized by strong basal ganglia inter-network connectivity. While this finding provided initial evidence for dynamic neural alterations in obesity, the study’s low-dimensional independent component parcellation (excluding some networks such as sensorimotor and visual networks) limits its scope. Thus, the relationship between BMI and whole-brain FC state dynamics remains largely unknown.

In addition to FC patterns, a complementary perspective on brain dynamics is offered by graph-theoretical network metrics. By computing topological properties (e.g., nodal efficiency) for each time window of the dFC, one can quantify the temporal variability of network topology. Recent studies suggest that the variability of such topological measures over time could serve as potential biomarkers for neurological and psychiatric conditions (16–18). These graph-based metrics capture system-level dynamic reconfigurations of the brain’s connectivity organization (19), providing a different vantage point than direct dFC variability. It is currently unknown, however, whether the temporal variability of network topological properties is related to BMI.

To address these gaps, the present study applied dFC analyses to a large resting-state fMRI dataset to examine (a) the association between BMI and the temporal properties of whole-brain FC states, and (b) the association between BMI and the temporal variability of network topological properties.

## 2 Materials and methods

### 2.1 Participants

Participants were drawn from the Human Connectome Project (HCP) 1,200 subject release (https://db.humanconnectome.org/data/projects/HCP_1200). The following exclusion criteria were applied: (a) missing BMI or demographic data (age, gender); (b) history of hyper/hypothyroidism or other endocrine disorders; (c) women who had recently given birth; (d) missing fMRI scanning session; (e) excessive head movement during scanning (mean frame-wise displacement > 0.25). The final sample consisted of 776 participants (age 22–37 years; males/females: 402/374; BMI range: 16.48–45.17). Informed consent was obtained from all participants, and the study protocol was approved by the WU institutional review board.

### 2.2 Data acquisition and preprocessing

Resting-state fMRI data were collected on a 3 T Siemens scanner using a standard 32-channel head coil. For each participant, four resting-state scans were acquired with the following parameters: repetition time (TR) = 720 ms; echo time (TE) = 33.1 ms; voxel size = 2 × 2 × 2 mm^3^; time points per scan = 2,400. We utilized the minimally preprocessed HCP dataset labeled “PTN” (Parcellation + Timeseries + Netmats), which has been used in recent dynamic brain studies [e.g., (20–22)]. Briefly, the HCP minimal preprocessing pipeline (23) was applied (including distortion correction and normalization to MNI-152 space using FreeSurfer), followed by the HCP “netmats” pipeline, which removes artifacts via ICA-FIX (24). Group independent component analysis (Group-ICA) was then performed using FSL’s MELODIC to derive group-level spatial IC maps and subject-specific time series for each IC.

### 2.3 The framework of dynamic functional network analysis

**Figure 1:**
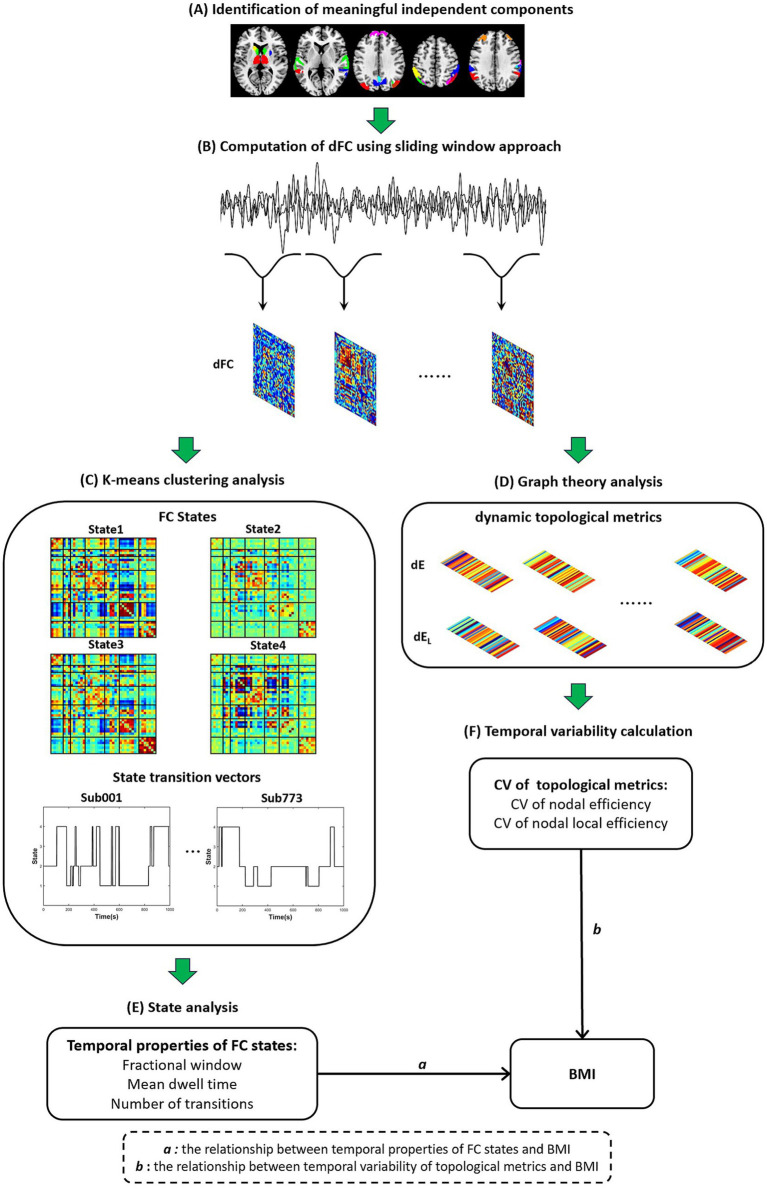
The analysis flowchart in the present study. **(A)** Identification of meaningful independent components. **(B)** Computation of dFC using sliding window approach. **(C)** K-means clustering analysis. **(D)** Graph theory analysis. **(E)** State analysis. **(F)** Temporal variability calculation.

The overall dynamic functional network analysis workflow is illustrated in Figure 1. First, meaningful ICs were identified based on their spatial patterns (see Section 2.4). Next, a sliding-window approach was used to compute dFC from the time series of the meaningful ICs (Section 2.5). The whole-brain dFC time series were then analyzed with k-means clustering to identify recurring FC states and their dynamics (Section 2.6). In parallel, dynamic graph theory metrics were computed from the dFC matrices, and their temporal variability was calculated (Section 2.7). Finally, statistical analysis was performed to examine the relationships between BMI and the temporal properties of FC states, and between BMI and the variability of topological properties (Section 2.8).

### 2.4 Identification of meaningful independent components

**Figure 2:**
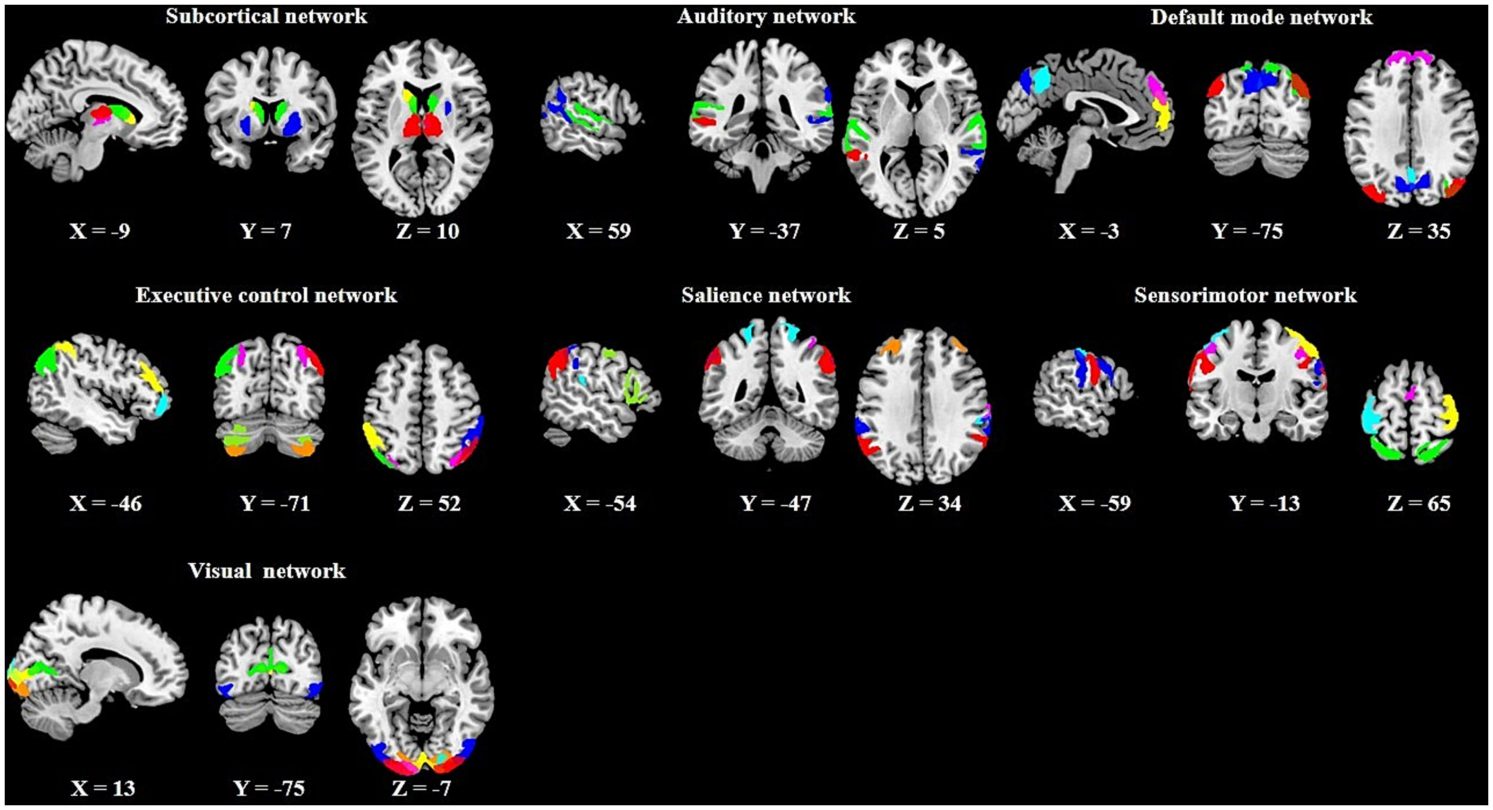
Spatial maps of the 43 meaningful independent components.

A total of 43 ICs were identified as meaningful functional components based on standard criteria (12): each IC’s spatial map had peak coordinates in gray matter and minimal overlap with white matter, ventricles, or other artifacts, and each time course exhibited a high dynamic range. Using a spatial correlation approach conducted by GIFT toolbox,^1^ we categorized these 43 ICs into seven canonical functional networks (based on a Stanford template): subcortical (SCN), auditory (AN), default mode (DMN), executive control (ECN), salience (SAN), sensorimotor (SMN), and visual (VN) (see Figure 2). Detailed information for each IC (brain regions, peak MNI coordinates, and spatial correlation with the template) is provided in Supplementary Table S1. The time series of the 43 ICs underwent the subsequent preprocessing steps to reduce physiological and scanner noise: removing the first 10 time points, detrending (regressing linear, quadratic, and cubic trends), despiking using 3D-DESPIKE, and low-pass filtering with frequency cut-off of 0.15 Hz.

### 2.5 dFC computation

The dFC of the 43 ICs were computed by using a sliding window approach, with the help of dFNC function in GIFT toolbox (See Footnote 1). The window type was set to a tapered window created by convolving a rectangle with a Gaussian (*σ* = 3TR). The window length was set to 70 TR (50.4 s) because previous study (7) suggested the window length between 30 and 60s can successfully capture the fluctuations of resting-state dFC. The window was slid step-wise by 1 TR, resulting in 2321 consecutive windows across the entire rfMRI scan. The FC matrices of each sliding window were computed from the inverse covariance matrix. To promote sparsity in estimations, the L1 norm penalty implemented in the LASSO framework (conducted based on the glasso function in GIFT toolbox) was applied on the covariance matrices, and the regularization parameter lambda *λ* was selected for each subject by cross-validation. All covariances were subsequently converted to correlation values and transformed into z-scores using Fisher r-to-z transformation. Eventually, a 43 (number of ICs) × 43 × 2,321 (number of windows) dFC matrix was derived for each participant representing the time-varying changes of FC along with the sliding windows.

### 2.6 Clustering analysis for FC states

The dFC matrices from all participants were concatenated and subjected to k-means clustering performed by using kmeans function^2^ in Matlab, to identify recurring FC patterns (i.e., FC states) and their temporal dynamics. In accordance with a series of dynamic brain state studies (12, 13, 15, 18, 20), we followed standard procedures to conduct clustering analysis. Specifically, to reduce redundancy between sliding windows as well as computational demand, windows corresponding to local maxima in FC variance were selected as subsamples for each subject. The k-means clustering algorithm was then applied to these subsampling windows using the L1 (Manhattan) distance as the similarity metric, and the clustering procedure was repeated 100 times to mitigate the bias introduced by random initialization of cluster centroids. To determine the optimal number of clusters, a cluster validity analysis based on the elbow criterion was conducted on subsampling windows from all subjects (65,910 windows in total; 83.93 ± 6.53 windows per subject), with the number of clusters (k) ranging from 2 to 10. Based on the elbow criterion, four clusters were identified as optimal. Using the resulting four cluster centroids as initialization, dFC data from all sliding windows across all participants were subsequently assigned to one of the four clusters (i.e., FC states) according to their similarity to the corresponding centroid. Thus, four FC states, and each participant’s sequence of FC states (state transition vector; Figure 1C) were obtained. For each participant, the state transition vector was then used to compute the following temporal properties of the FC states: fractional window (FW), mean dwell time (MDT), and number of transitions (NT). FW (in %) is the proportion of time windows spent in each state. MDT is the average length of consecutive windows spent in a given state before transitioning to a different state (i.e., the mean duration of uninterrupted occupancy of that state). NT is the total count of transitions from one state to another across the entire 2,321-window sequence for that participant.

To assess whether the FC patterns and their BMI relevance were stable across different clustering number, we additionally examined alternative number of clusters (k = 3, 5 and 6), following the same procedures as the primary analysis. These results were mainly presented in Supplementary Material.

### 2.7 Dynamic topological properties analysis

The dFC matrices were also used to compute dynamic topological properties based on graph theory analysis, using GRETNA toolbox.^3^ The 43 × 43 FC matrices in each window were firstly binarized respect to a set of fixed sparsity threshold. We then set the threshold as a range of sparsity level instead of one single value, and computed the area under the curve (AUC) of each network metric across sparsity levels. We focused on two network topological properties: efficiency and local efficiency. Efficiency of each node (IC) is computed as the reciprocal of the shortest path lengths from the node to all other nodes in the network, which represents the parallel information transfer capacity of that node in the network. Local efficiency of a node is defined as the global efficiency of the sub-network containing the node and its all direct neighbors, which measures how efficient the communication is among the first neighbors of this node when it is removed. For each participant, we calculated the coefficient of variation (CV = SD/mean) of the dynamic topological properties as the temporal variability of topological metrics (17, 18).

### 2.8 Statistical analysis

We adopted Pearson correlation (controlling for the effects of age and gender) to examine the associations between BMI and temporal properties of FC states, and between BMI and temporal variability of topological metrics. For those correlations involve multiple states or ICs (FW and MDT for each FC state, CV of nodal efficiency and local efficiency for each IC), the *p*-values of these correlations were corrected by FDR (False Discovery Rate) method, which were marked as *pcorr* in the Results section.

## 3 Results

### 3.1 The clustered FC states

**Figure 3:**
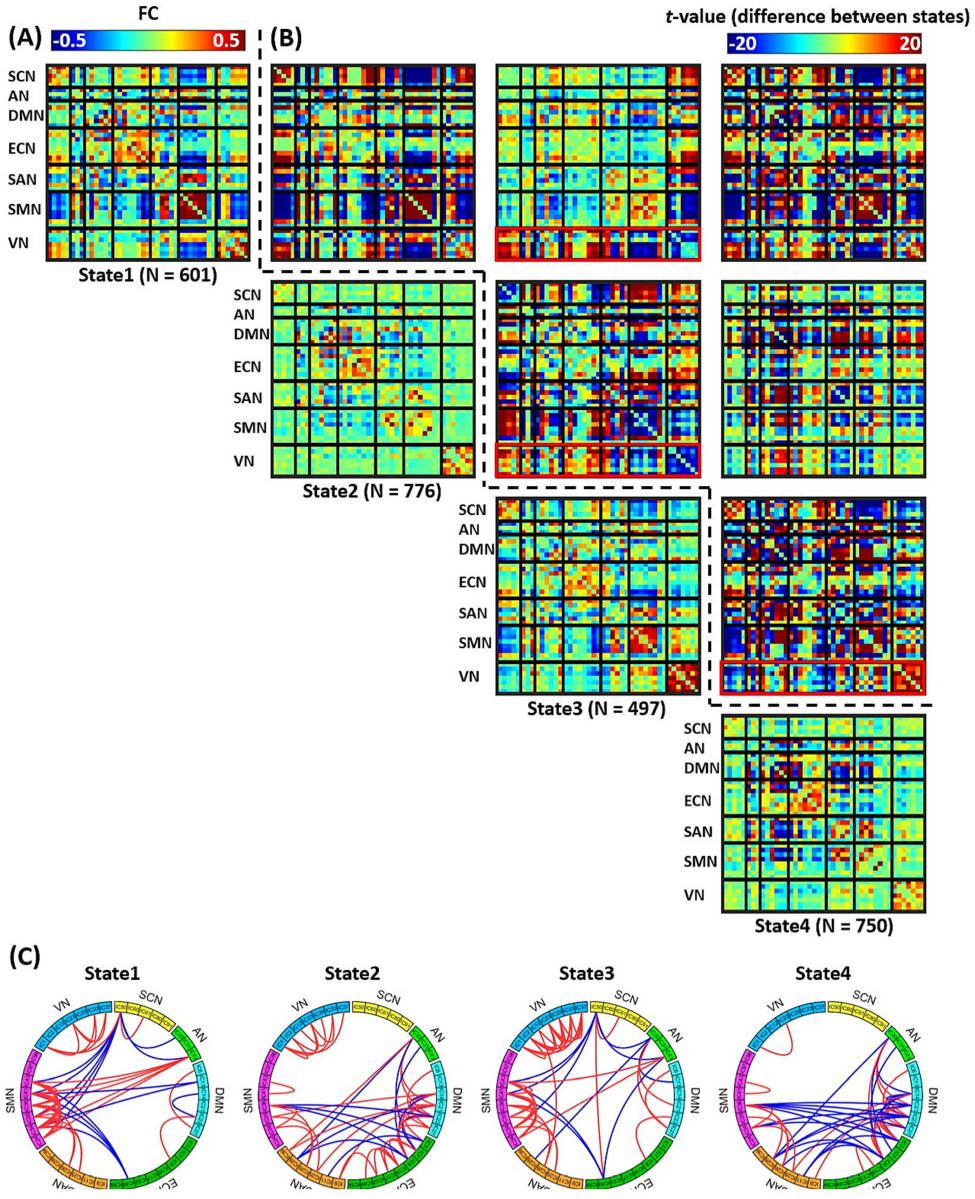
**(A)** Diagonal plots: the FC states derived by *k*-means clustering, N indicate the numbers of participants that have valid time windows in each state. **(B)** Off-diagonal plots: FC differences between each pair of states. **(C)** The top 5% strongest FC connections of each state.

The clustered four FC states were visualized in Figure 3A, and the FC states for groups of healthy weight (BMI 18.5–24.9), overweight (BMI 25–29.9), and obesity (BMI ≥ 30) were calculated by averaging subject-specific centroids of each group (see Supplementary Figure S3). The high intra-class correlations (Supplementary Table S2) for FC states among these groups indicated high BMI-related consistency in FC states. The differences between each pair of FC states were presented in Figure 3B, and the top 5% strongest connections of each state were also presented in Figure 3C. It can be observed these FC states differed significantly from each other. In the overall level, state 1 had the strongest averaged FC strength while that of state 2 was weakest (see Supplementary Figure S1A). In the regional level, we found state 2 and state 4 exhibited high pattern similarity (see Supplementary Figure S1B) whose strong FCs were mainly concentrated within/between regions of DMN, ECN and SAN. State 1 and state 3 also had high pattern similarity (Supplementary Figure S1B) whose strong FCs, unlike state 2 and state 4, were mainly concentrated within SMN and between SMN and SCN. There were also distinguished differences between the states with high pattern similarity. For example, the FC pattern of state 3 differed significantly from state 1 in both intra-network and inter-network connections of VN. Specifically, state 3 had stronger positive intra-network FC in VN, negatively decreased internetwork FC of VN-SCN and VN-ECN, and positively increased internetwork FC of VN-SMN (see red frame in the plot of first row and third column, Figure 3B). Such differences also existed when compared with state 2 and state 4 (red frame in the plots of second row, third column and third row, forth column, Figure 3B).

### 3.2 The relationship between temporal properties of FC states and BMI

**Figure 4:**
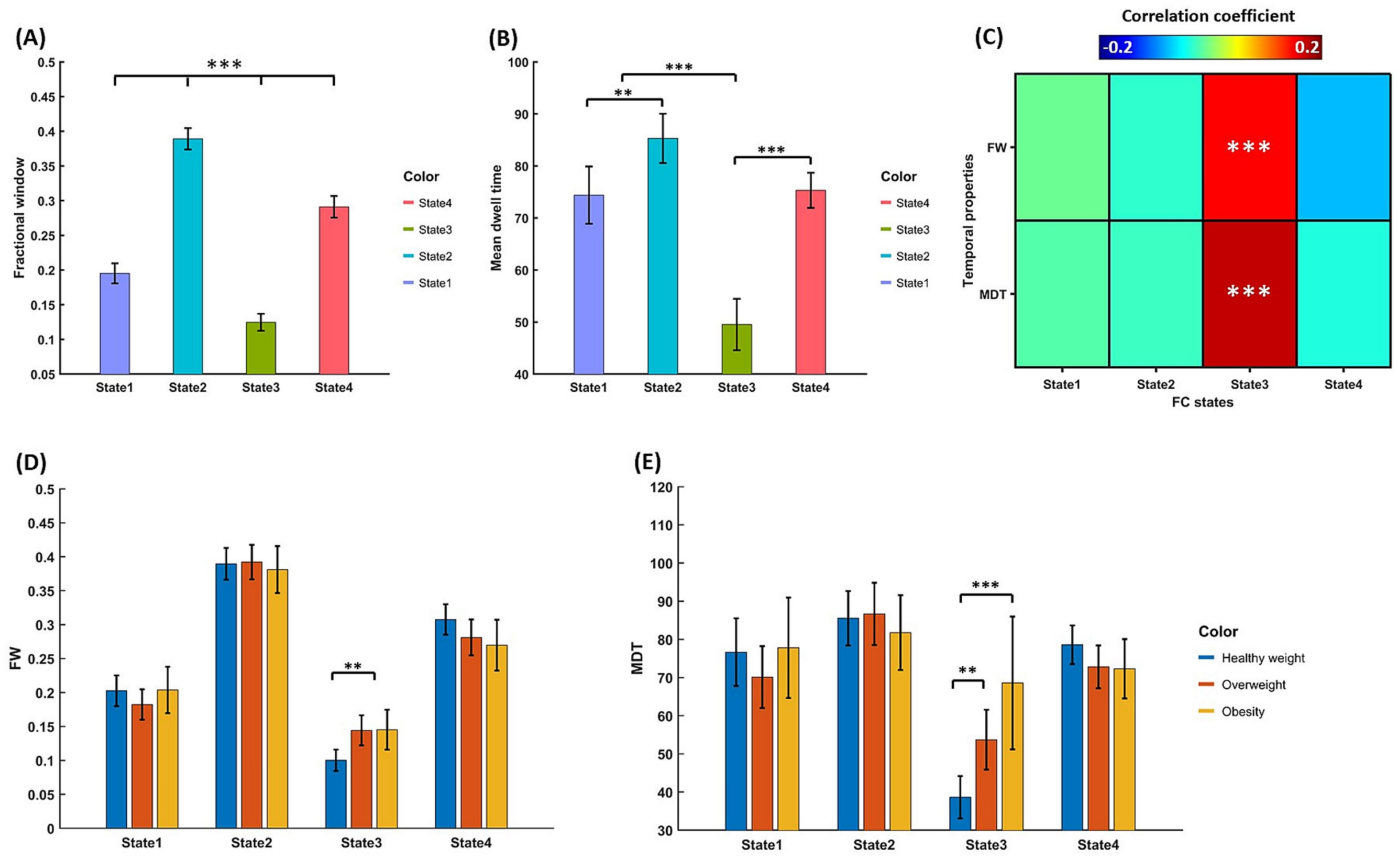
**(A)** FW and **(B)** MDT of different FC states. **(C)** The correlation coefficients between FW and MDT of FC states and BMI. **(D)** FW and **(E)** MDT of each FC state across groups of healthy weight (BMI 18.5–24.9), overweight (BMI 25–29.9), and obesity (BMI ≥ 30).

The FW and MDT of each FC state were displayed in Figures 4A,B. It can be observed that FW and MDT of state 2 were highest among FC states, while those of state 3 were lowest among all states. Importantly, we identified significant positive correlations between FW of state 3 and BMI (*r* = 0.15, *pcorr* < 0.001; Figure 4C), and between MDT of state 3 and BMI (*r* = 0.17, *pcorr* < 0.001; Figure 4C). No significant correlation was found between temporal properties of other states and BMI. In line with the positive BMI-related association of state 3, we found the FW and MDT of state 3 in healthy weight group were lower than those in overweight (FW: *t* = −3.26, *pcorr* < 0.01; MDT: *t* = −3.16, *pcorr* < 0.01) and obesity (FW: *t* = −2.85, *pcorr* < 0.01; MDT: *t* = −4.21, *pcorr* < 0.001) groups, which were presented in Figures 4D,E.

To assess the robustness of this finding, we repeated the dFC state estimation using alternative clustering number (k = 3, 5 and 6). We found that the VN-dominant connectivity pattern was largely preserved when using k = 5 and k = 6 (Supplementary Figures S4B,C), and the association between the VN-dominant state and BMI remained similar across these clustering numbers (Supplementary Figures S5B,C). However, when using k = 3, the VN-dominant connectivity pattern was less clearly separated from other connectivity configurations (Supplementary Figure S4A), and the association between FC state and BMI was not statistically significant (Supplementary Figure S5A).

### 3.3 The relationship between temporal variability of topological metrics and BMI

**Figure 5:**
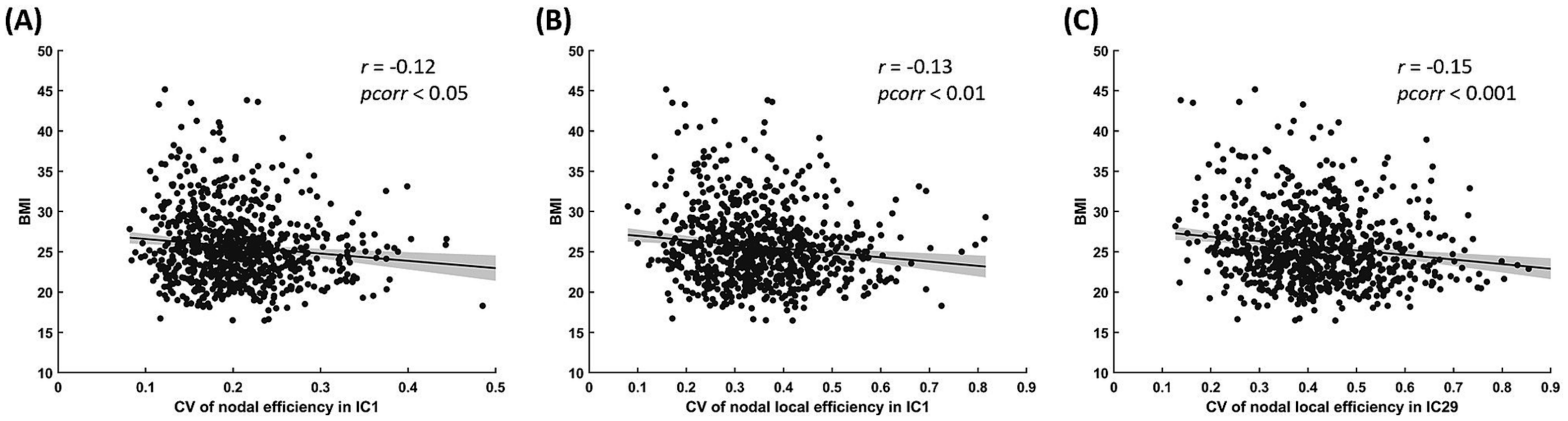
The relationships between CV of nodal efficiency in IC 1 (L-MOG and B-IOG) and BMI **(A)**, between CV of nodal local efficiency in IC 1 and BMI **(B)**, and between the CV of nodal local efficiency in IC 29 (B-CAL) and BMI **(C)**. All shown correlations are significant after FDR correction (i.e., *p_corr_* < 0.05).

We found the significant negative correlations between CV of nodal efficiency in IC 1 (left middle occipital gyrus, L-MOG, and, bilateral inferior occipital gyrus, B-IOG) and BMI (*r* = −0.12, *pcorr* < 0.05; Figure 5A), between CV of nodal local efficiency in IC 1 (*r* = −0.13, *pcorr* < 0.01; Figure 5B) and BMI, and between the CV of nodal local efficiency in IC 29 (bilateral calcarine sulcus, B-CAL) and BMI (*r* = −0.15, *pcorr* < 0.001; Figure 5C).

## 4 Discussion

In this study, we investigated the association between BMI and the dynamics of brain functional networks. We identified four FC states with distinguished network configurations. Notably, one of these states (state 3), characterized by strong positive intra-network FC of VN, strong positive inter-network FC of VN-SMN and strong negative inter-network FC of VN-SCN and VN-ECN, its temporal properties (FW and MDT) were positively correlated with BMI. Negative correlations were found between BMI and variability of topological properties in the ICs belonging to VN. These findings provide novel evidence of BMI-related functional network dynamics, but the observed associations are modest and should be interpreted as exploratory, providing a large-sample foundation for future nutrition-focused studies that incorporate more specific metabolic or dietary measures beyond BMI.

### 4.1 The association between BMI and dynamics of FC states

The significant correlations between BMI and the temporal properties (FW and MDT) of FC state 3 suggest that this specific whole-brain connectivity configuration is highly relevant to obesity. State 3 is distinguished by stronger positive intra-network connectivity in the VN and between the VN and SMN, along with more negative (or weaker) inter-network connectivity between the VN and SCN/ECN. This profile underscores the important roles of the VN and its interactions with other networks (particularly the SCN, ECN, and SMN) in the neurophysiology of obesity. The VN and SMN are involved in processing external visual cues (including food-related stimuli), forming a critical interface between food cues and motivated behavior in obesity (25, 26). Meanwhile, the SCN and ECN are key components of the brain’s reward circuitry (27) and are involved in cognitive control processes such as inhibitory control (28).

Considering the positive correlation we observed between the FW of state 3 and BMI, we speculate that obesity may be associated with increased functional connectivity within the VN and between the VN and SMN, and concurrently decreased connectivity between the VN and the SCN/ECN. Such a pattern could imply enhanced sensory salience of food-related cues coupled with an impaired ability to suppress or ignore those cues. This speculation is supported by prior findings: higher BMI has been associated with heightened connectivity within visual regions (29, 30) and between the VN and SMN (31), as well as with reduced connectivity between visual and salience/control regions—patterns also observed in addictions that share neurobiological overlaps with obesity (32, 33).

Our findings further suggest that VN-related functional connectivity changes in obesity may not be uniformly present throughout the entire resting period, but rather emerge because individuals with higher BMI spend disproportionately more time in a particular state (state 3) that has a distinct VN-centric connectivity pattern. The positive correlation between BMI and the MDT of state 3 indicates that higher-BMI individuals remain in this state for longer durations and have more difficulty transitioning to other states, reflecting a reduction in flexibility of brain state switching (34). This reduced flexibility likely leads to lower variability in connectivity over time, especially for the VN-related connections that characterize state 3. Therefore, we propose a speculation whereby higher BMI is accompanied with prolonged occupancy of the VN-characterized state 3 (i.e., reduced state transition flexibility), which in turn results in diminished temporal variability of connectivity in VN. This speculation is supported by one recent observation of altered dFC variability in VN regions among individuals with higher BMI (11), and by our finding of reduced variability of topological properties in the VN with higher BMI.

The robustness analysis using alternative clustering numbers further supports the stability of the identified VN-dominant state. Specifically, similar connectivity patterns and brain–BMI associations were observed when using k = 5 and k = 6. In contrast, the association was not statistically significant when using k = 3. One possible explanation is that specifying a smaller number of clusters may merge multiple connectivity configurations into broader states, thereby reducing the ability to isolate specific connectivity patterns. In state-based dFC analyses, clustering methods group time-resolved connectivity matrices into a set of recurring connectivity states, and the number of clusters determines the granularity at which these states are represented. When the model order is too low, heterogeneous connectivity patterns may be combined into a single state, potentially obscuring biologically meaningful distinctions between connectivity configurations. Previous studies have similarly emphasized that the choice of cluster number influences the resolution and interpretability of connectivity states in dFC analyses (7, 12, 35). Taken together, these findings suggest that the observed association is robust across reasonable clustering solutions, and that the non-significant result at k = 3 likely reflects reduced state resolution rather than the absence of the underlying connectivity–behavior relationship.

### 4.2 The association between BMI and variability of topological properties

We found significant negative correlations between BMI and the temporal variability of nodal efficiency and local efficiency in ICs of the VN, suggesting modest, population-level associations between higher BMI and lower temporal variability of these network efficiency measures. In other words, higher BMI was linked to a more stable efficiency profile in visual network nodes over time. Reduced variability in nodal efficiency suggests that those VN regions in individuals with obesity exhibit less flexible communication with the rest of the brain, consistent with the above-mentioned findings on regional FC variability in the VN (11). Similarly, reduced variability in local efficiency implies a more rigid pattern of interactions between VN regions and their immediate neighbors, pointing to diminished adaptability in local network processing. Prior work has noted that brain regions with very strong functional connectivity are less easily modulated (12, 36). In this context, the reduced flexibility we observed could be a consequence of the strong intra-VN connectivity characterizing state 3 and the extended time spent in that state by individuals with obesity. This interpretation is in line with the idea that obese individuals have a more rigid visual system, which may limit efficient integration of sensory information.

Moreover, our findings help pinpoint specific brain regions implicated in these effects. The ICs that showed reduced efficiency variability (IC 1 and IC 29) correspond to occipital regions—namely, the left middle occipital gyrus (L-MOG), bilateral inferior occipital gyrus (B-IOG), and bilateral calcarine sulcus (B-CAL) (5, 37). These regions are core nodes of the VN, supporting both basic visual processing and higher-order visual functions. The MOG is primarily involved in the integration of visual features, the IOG contributes to object recognition and visual attention, and the CAL (primary visual cortex) handles early-stage visual processing (38–40). Dysfunction or inflexible activity in these regions could alter how food-related visual cues are processed, potentially contributing to maladaptive eating behaviors in obesity. Empirical evidence supports this notion: obese individuals exhibit abnormal occipital responses to food cues, including heightened activation of visual cortex and disrupted connectivity between occipital areas and reward/salience circuits (25, 41, 42). Furthermore, these VN regions are involved in executive functions; effective communication between occipital (visual) regions and control networks is critical for inhibiting automatic responses to salient food cues and maintaining goal-directed behavior (11, 43). Given that obesity is often accompanied by deficits in executive function (e.g., impairments in inhibitory control and cognitive flexibility) (44, 45), our findings point to a dual role of the VN in both heightened sensory-driven reactivity and compromised regulatory control in obesity. This highlights the VN as a key neural substrate linking sensory cue reactivity and executive control deficits in the neurobiological mechanisms of obesity.

### 4.3 Future directions and limitations

Although our study provides novel insights into the association between BMI and brain network dynamics, several limitations should be noted. First, because our data are cross-sectional, we could not determine causal relationships between BMI and dynamic functional connectivity. Longitudinal studies will be important in the future to establish causality (e.g., whether changes in BMI lead to changes in network dynamics or vice versa). Second, we did not directly assess cognitive functions (such as executive function) in relation to BMI and network dynamics, even though we speculate they play a role. Given the known links between cognitive function and both obesity (45–47) and dFC characteristics (20), future work should include behavioral measures of cognitive performance to elucidate how cognitive factors might mediate or moderate the neural differences observed. Third, our sample consisted of young adults within a relatively narrow age range (22–37 years). It remains to be seen whether the observed BMI-related dynamic connectivity patterns hold across other age groups. Future studies could apply our analytical framework to cohorts with broader age ranges to test the generalizability and developmental course of these findings. Fourth, The FC states in the present study were derived from concatenated time windows across all participants, following a commonly used framework in dynamic functional connectivity studies (12, 13). This group-level clustering approach identifies a set of representative connectivity states that provide a common reference space for characterizing individual temporal dynamics. Nevertheless, this framework implicitly assumes that a shared set of states can capture the major patterns of functional connectivity across individuals, and therefore may not fully account for inter-individual variability in brain state organization, despite we observed that these FC states were highly consistent across groups with different level of BMI. Future studies should consider subject-specific or hierarchical approaches to capture individual variability in state expression.

## 5 Conclusion

Our study reveals the relationship between BMI and the dynamics of brain functional networks. We found that BMI is positively correlated with temporal properties (FW and MDT) of a specific FC state (state 3) characterized by distinctive intra-network and inter-network connectivity of the VN. We also found that BMI is negatively correlated with the temporal variability of network topological properties in VN regions. Taken together, these results suggest that individuals with higher BMI have less flexible functional network dynamics (spending more time in a particular VN-related state) accompanied by reduced variability in the brain’s topological organization. However, the modest effect sizes indicate limited explanatory power and warrant cautious interpretation and future validation. These findings advance our understanding of the links between BMI and brain network dynamics, and highlight the role of VN in the neurophysiological mechanisms of obesity.

## Data Availability

The original contributions presented in the study are included in the article/Supplementary material, further inquiries can be directed to the corresponding author.

## Footnotes

1
mialab.mrn.org/software/gift

2
https://www.mathworks.com/help/stats/kmeans.html

3
http://www.nitrc.org/projects/gretna
