# Supplementary material for: Characterizing the relationship between functional network dynamics and the body mass index
Source: Front Nutr. 2026 Apr 14;13:1734850. doi: 10.3389/fnut.2026.1734850 (PMC13120893; doi:10.3389/fnut.2026.1734850)
Supplement: Supplementary file 1 [file Table_1.docx]

Supplementary Material


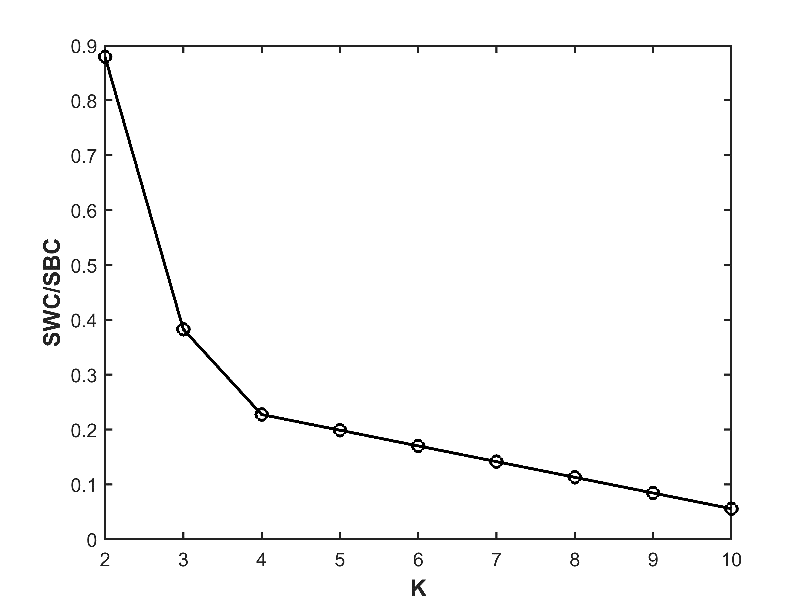


**Figure S1.** The visualized results of cluster number validity analysis using elbow criteria. SWC and SBC represent the sum of within cluster distance and the sum of between cluster distance respectively.


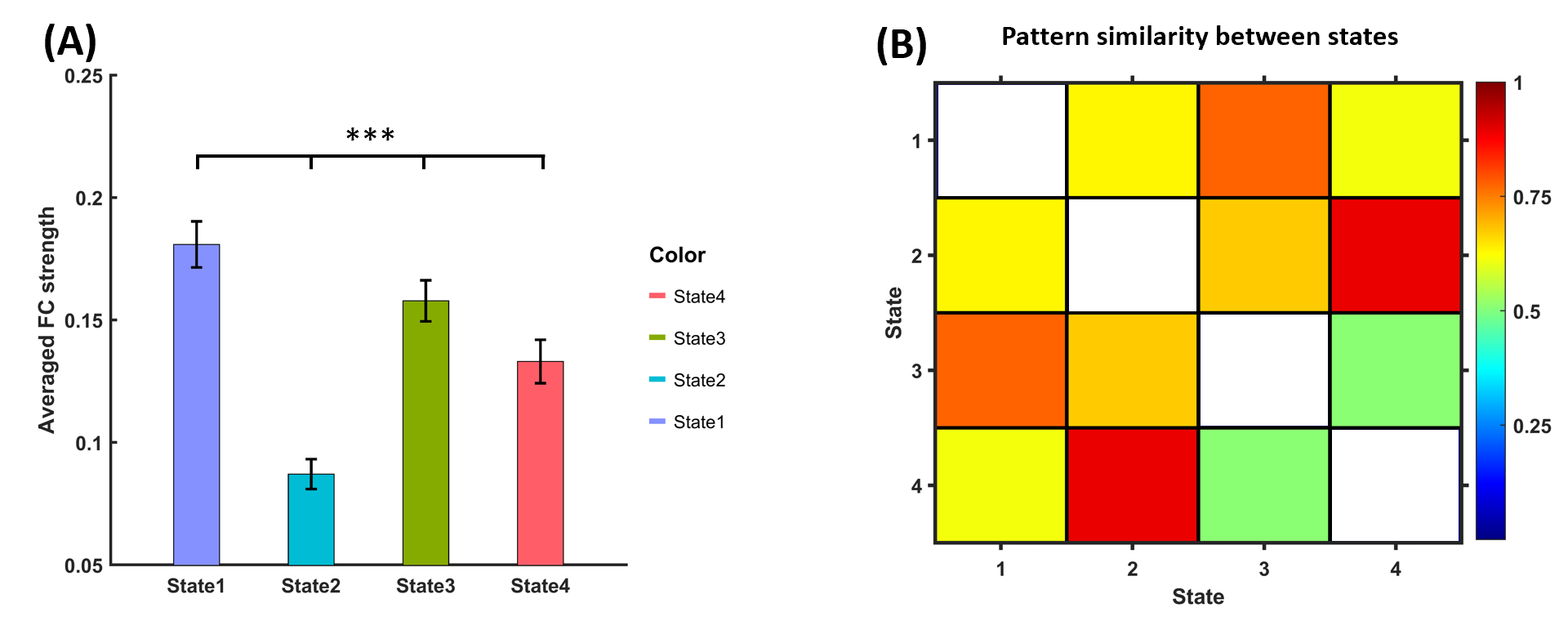
**Figure S2.** (A) Averaged FC strength of different FC states, *** representing *pcorr* < 0.001 (paired-sample *t* test, FDR corrected). (B) Similarity matrix of the FC pattern between different states.


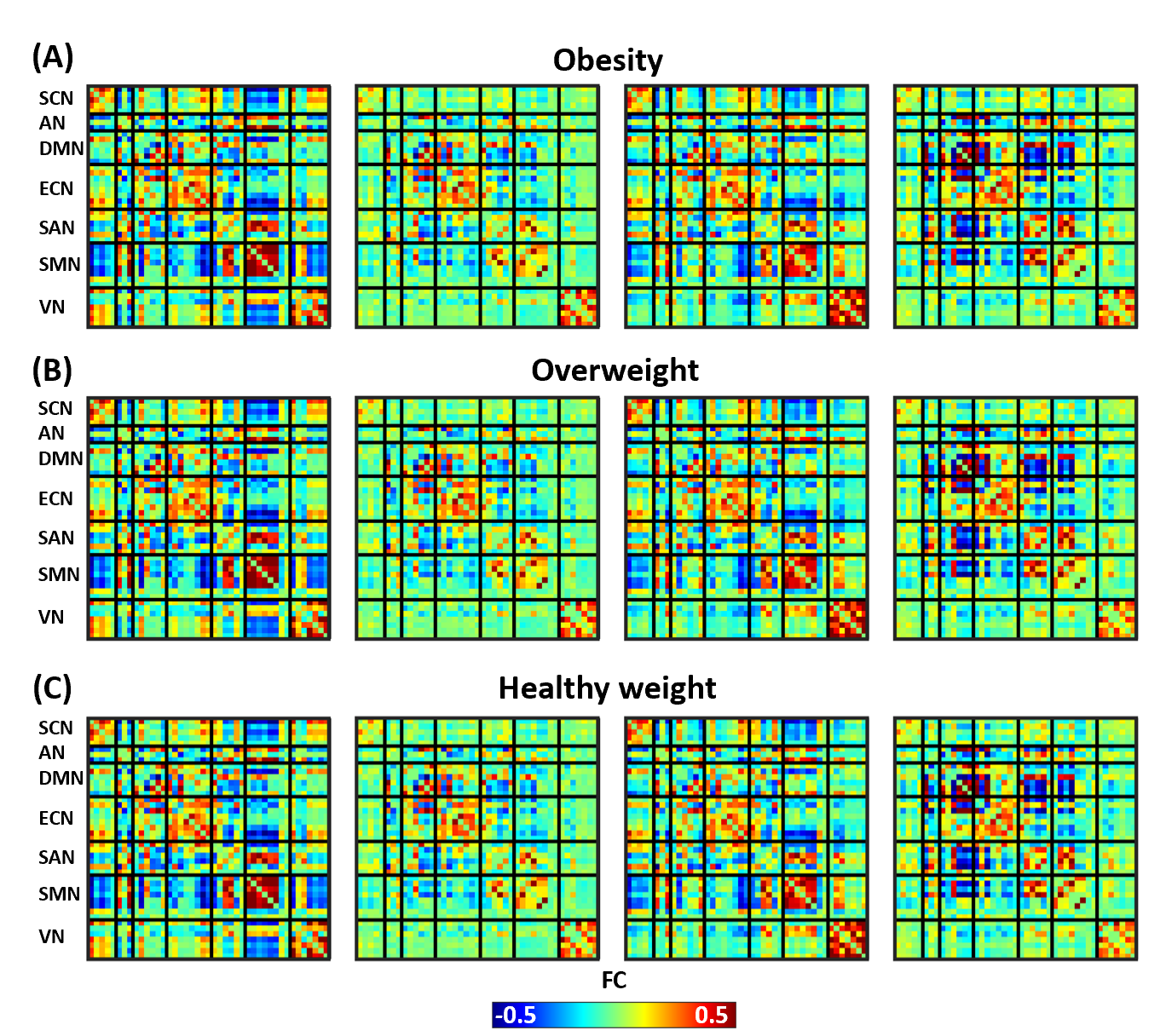
**Figure S3.** FC states in different groups of participants: (A) obesity (BMI ≥ 30); (B) overweight (BMI 25-29.9); (C) healthy weight (BMI 18.5-24.9).


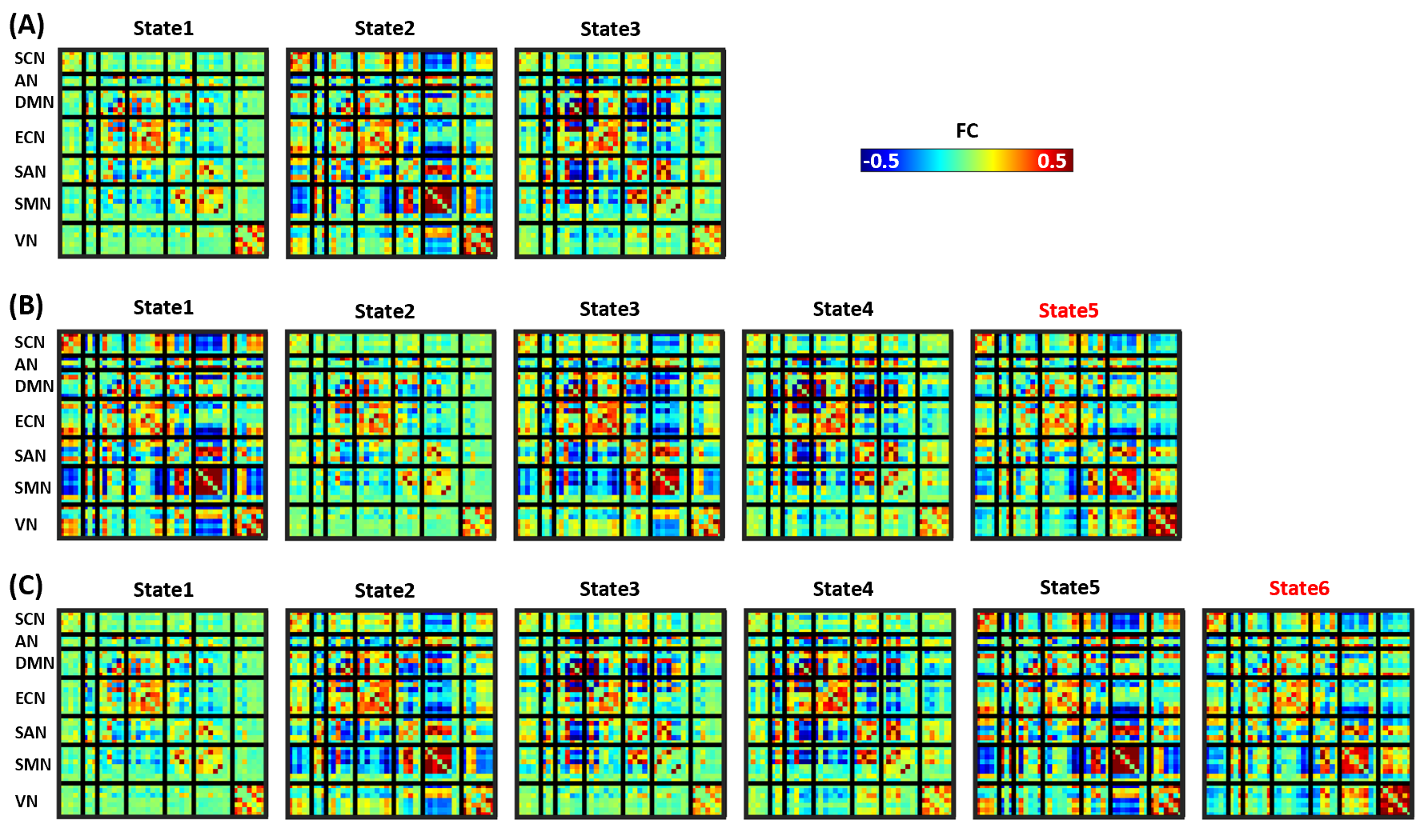
 **Figure S4.** FC states derived using alternative clustering numbers: (A) k = 3; (B) k = 5; (C) k = 6. Note: State 5 in (B) and State 6 in (C) are the VN-dominant states similar to State 3 in Figure 3.


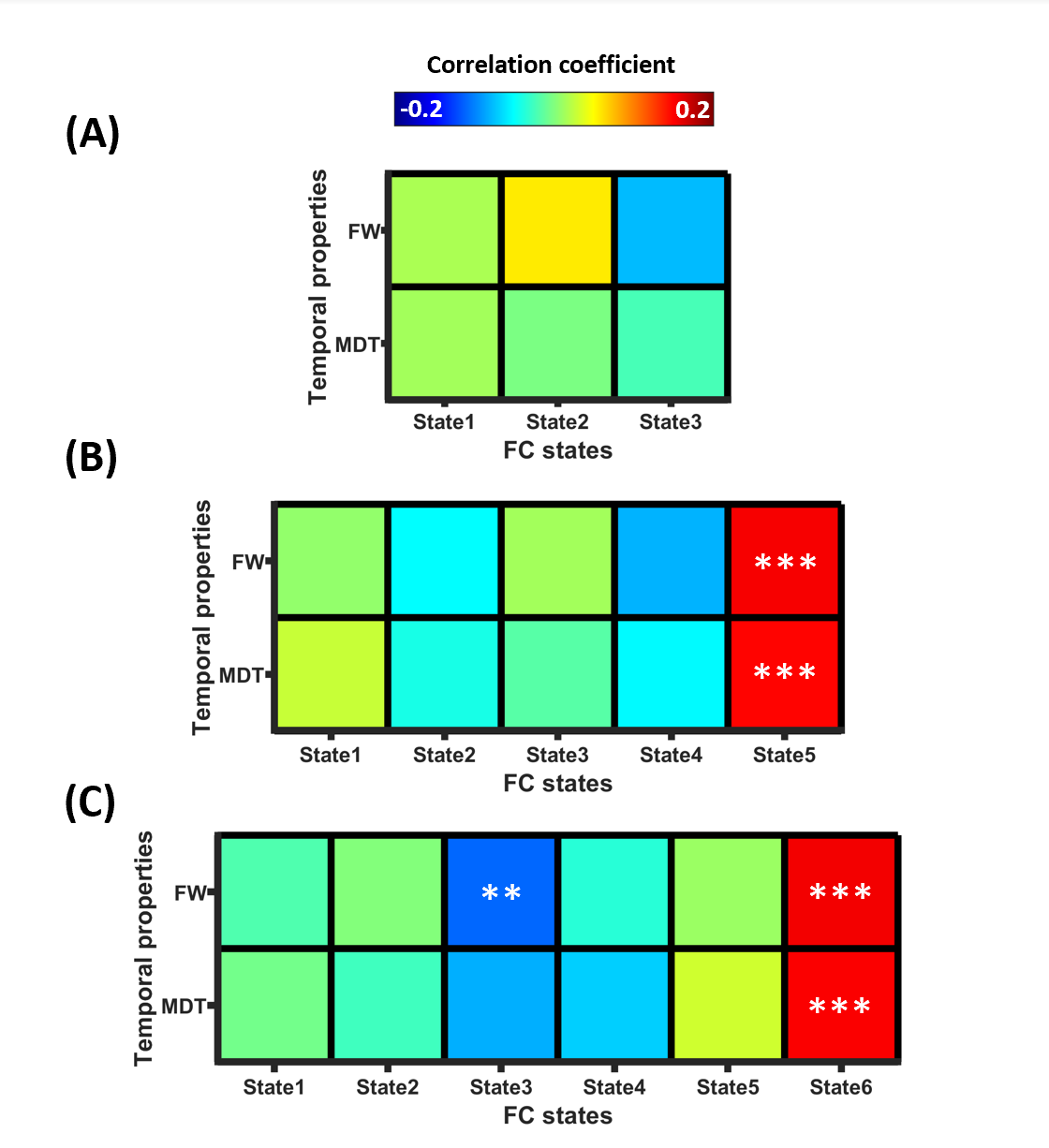


**Figure S5.** The correlation coefficients between FW and MDT of FC states and BMI using alternative clustering numbers: (A) k = 3; (B) k = 5; (C) k = 6. Note: ** and *** represent *pcorr* < 0.01 and *pcorr* < 0.001 respectively.

**Table S1.** Detailed information of the meaningful ICs

| IC Index | Brain area (AAL atlas) | peak MNI coordinates of spatial maps | | | *r^[[1]](#footnote-1)^* |
| --- | --- | --- | --- | --- | --- |
|  |  | x | y | z |  |
| Subcortical network (SCN) | | | | | |
| 1. IC 50 | Thalamus_B | 12 | -13 | 10 | 0.43 |
| 1. IC 60 | Putamen_R | 24 | 10 | -2 | 0.15 |
|  | Putamen_L | -24 | 10 | -7 |  |
| 1. IC 61 | Caudate_R | 10 | 0 | 14 | 0.27 |
|  | Caudate_L | -7 | 2 | 10 |  |
| 1. IC 80 | Thalamus_B | 3 | -3 | 2 | 0.24 |
| 1. IC 91 | Caudate_L | -14 | 16 | 6 | 0.16 |
| Auditory network (AN) | | | | | |
| 1. IC 20 | Temporal_Mid_L | -47 | -38 | 0 | 0.25 |
| 1. IC 31 | Temporal_Mid_R/SupraMarginal_R | 64 | -46 | 26 | 0.20 |
| 1. IC 43 | Temporal_Sup_R | 66 | -20 | 13 | 0.39 |
|  | Temporal_Sup_L | -64 | -20 | 12 |  |
| Default mode network (DMN) | | | | | |
| 1. IC 6 | Occipital_Mid_L | -34 | -82 | 40 | 0.53 |
|  | Occipital_Mid_R/Angular_R | 43 | -76 | 36 |  |
| 1. IC 13 | Precuneus_B/Cuneus_B | 10 | -76 | 40 | 0.73 |
| 1. IC 14 | Occipital_Mid_R/Parietal_Sup_R/  Precuneus_R | 42 | -80 | 32 | 0.33 |
|  | Precuneus_L | -12 | -74 | 52 |  |
| 1. IC 21 | Frontal_Sup_Medial_B/ Frontal_Sup_B | -13 | 57 | 30 | 0.32 |
| 1. IC 22 | Frontal_Sup_Medial_B | 2 | 60 | 16 | 0.65 |
| 1. IC 27 | Temporal_Mid_R/Angular_R | 60 | -57 | 20 | 0.22 |
|  | Temporal_Mid_L/Angular_L | -53 | -57 | 20 |  |
|  | Precuneus_B | 0 | -62 | 43 |  |
| executive control network (ECN) | | | | | |
| 1. IC 3 | Angular_R/Parietal_Inf_R | 54 | -64 | 36 | 0.27 |
| 1. IC 7 | Parietal_Inf_R/SupraMarginal_R | 57 | -36 | 50 | 0.31 |
| 1. IC 8 | Angular_L/Parietal_Inf_L | -46 | -67 | 44 | 0.29 |
| 1. IC 16 | Temporal_Inf_L/Temporal_Mid_L | -56 | -46 | -12 | 0.26 |
|  | Temporal_Mid_R/Temporal_Inf_R | 62 | -43 | -10 |  |
|  | Parietal_Sup_L/Parietal_Inf_L | -26 | -74 | 47 |  |
|  | Angular_R/Parietal_Sup_R | 38 | -68 | 501 |  |
| 1. IC 17 | Frontal_Mid_L/Frontal_Inf_Tri_L | -47 | 34 | 24 | 0.29 |
|  | Parietal_Inf_L | -52 | -47 | 50 |  |
| 1. IC 32 | Frontal_Mid_L/Frontal_Mid_Orb_L | -43 | 52 | 2 | 0.27 |
|  | Frontal_Mid_R/Frontal_Mid_Orb_R | 42 | 56 | 0 |  |
| 1. IC 49 | Cerebelum_Crus2_R | 30 | -72 | -47 | 0.26 |
|  | Cerebelum_Crus2_L | -30 | -72 | -47 |  |
| 1. IC 59 | Cerebelum_Crus2_L/Cerebelum_Crus1_L | -32 | -72 | -38 | 0.25 |
|  | Cerebelum_Crus2_R/Cerebelum_Crus1_R | 34 | -70 | -40 |  |
| Salience network (SAN) | | | | | |
| 1. IC 9 | Parietal_Inf_L/Angular_L | -57 | -52 | 40 | 0.27 |
|  | Parietal_Inf_R/SupraMarginal_R | 57 | -50 | 44 |  |
| 1. IC 11 | Parietal_Inf_L/SupraMarginal_L | -60 | -36 | 44 | 0.51 |
|  | SupraMarginal_R | 62 | -38 | 42 |  |
| 1. IC 25 | Postcentral_R/SupraMarginal_R | 57 | -22 | 50 | 0.19 |
|  | Temporal_Inf_R | 54 | -60 | -10 |  |
| 1. IC 26 | SupraMarginal_L/Temporal_Sup_L | -60 | -30 | 18 | 0.31 |
|  | SupraMarginal_R | 50 | -30 | 28 |  |
|  | Parietal_Sup_L/Precuneus_L | -18 | -50 | 64 |  |
|  | Postcentral_R/Parietal_Sup_R | 20 | -50 | 70 |  |
| 1. IC 28 | Frontal_Mid_L | -30 | 52 | 26 | 0.43 |
|  | Frontal_Mid_R | 32 | 52 | 28 |  |
| 1. IC 39 | Frontal_Inf_Tri_L/Frontal_Inf_Oper_L/Frontal_Inf_Orb_L | -47 | 20 | -6 | 0.23 |
|  | Precentral_L | -50 | 2 | 50 |  |
|  | Supp_Motor_Area_L | -2 | 6 | 66 |  |
| Sensorimotor network (SMN) | | | | | |
| 1. IC 24 | Postcentral_L | -53 | -7 | 24 | 0.15 |
|  | Postcentral_R | 53 | -6 | 24 |  |
| 1. IC 34 | Postcentral_L/ Parietal_Inf_L | -62 | -18 | 30 | 0.12 |
|  | Postcentral_R | 57 | -13 | 30 |  |
|  | Precentral_L | -60 | 4 | 30 |  |
| 1. IC 35 | Parietal_Sup_R | 20 | -64 | 64 | 0.15 |
|  | Parietal_Sup_L | -20 | -62 | 64 |  |
| 1. IC 36 | Postcentral_L | -38 | -16 | 46 | 0.26 |
|  | Precentral_R | 50 | -7 | 54 |  |
|  | Supp_Motor_Area_B | 4 | 2 | 62 |  |
| 1. IC 41 | Postcentral_R/Precentral_R | 42 | -22 | 64 | 0.32 |
| 1. IC 42 | Postcentral_L/Precentral_L | -37 | -24 | 66 | 0.30 |
| 1. IC 97 | Vermis_4_5/Cerebelum_4_5_B | 8 | -52 | -8 | 0.29 |
| 1. IC 98 | Cerebelum_4_5_R/Cerebelum_6_R | 16 | -56 | -16 | 0.22 |
|  | Cerebelum_4_5_L/Cerebelum_6_L | -18 | -56 | -16 |  |
| Visual Network (VN) | | | | | |
| 1. IC 1 | Occipital_Mid_L/ Occipital_Inf_L | -26 | -100 | -6 | 0.27 |
|  | Occipital_Inf_R/Lingual_R/Calcarine_R | 28 | -98 | -6 |  |
| 1. IC 2 | Occipital_Mid_L/Occipital_Inf_L | -36 | -92 | 3 | 0.56 |
|  | Occipital_Inf_R/Occipital_Mid_R | 40 | -87 | -2 |  |
| 1. IC 12 | Calcarine_B | 10 | -72 | 13 | 0.57 |
| 1. IC 23 | Occipital_Mid_L | -24 | -96 | 2 | 0.26 |
| 1. IC 29 | Calcarine_B | -7 | -93 | -2 | 0.30 |
| 1. IC 33 | Occipital_Mid_R/Calcarine_R | 26 | -98 | 10 | 0.41 |
| 1. IC 37 | Lingual_L/Calcarine_L | -10 | -94 | -12 | 0.26 |
|  | Lingual_R | 14 | -92 | -6 |  |

Abbreviations: IC, independent component; L, left; R, right; Ant, anterior; Inf, inferior; Med, medial; Mid, middle; Sup, superior; Supp, supplementary; Oper, opercularis; Orb, orbital; Tri, tri

**Table S2.** ICC (intra-class correlation) for each FC state among groups of participants (i.e., healthy weight, overweight, and obesity)

| FC State | State 1 | State 2 | State 3 | State 4 |
| --- | --- | --- | --- | --- |
| ICC | 0.93 | 0.94 | 0.93 | 0.94 |

1. The parameter *r* refers to the spatial correlation coefficient between each IC and a specific network of the Stanford functional ROI template ([findlab.stanford.edu/functional_ROIs.html](http://findlab.stanford.edu/functional_ROIs.html)). [↑](#footnote-ref-1)
